# Supplementary material for: Uracil-driven ROS signaling and larval TrpA1-B neuron activation are required for priming the adult Drosophila gustatory response to bacteria
Source: PLoS Biol. 2026 Oct 5;24(10):e3004042. doi: 10.1371/journal.pbio.3004042 (PMC13657326; doi:10.1371/journal.pbio.3004042)
Supplement: Supplementary file 1 [file pbio.3004042.s013.pdf]

Dear PLoS Biology editors, Dear reviewers,

Please find the point-to-point response to the reviewers ‘comments and requests.

Best regards,
Julien Royet

Text color significance.
Green: Reviewers comments and requests
Blue: Our responses
Red: Modifications in the text

Reviewer #1:

1. In the abstract: The sentence "Animals perceive their environment through their sensory systems why rely on neuronal circuits and proteins, but can be modulated by internal physiological conditions or exogenous factors" is grammatically very awkward. The phrase "why rely on" is likely an error, and it should probably read "which rely on."

The mentioned text has been changed as follow.

Animals perceive their immediate environment through their sensory systems. While these systems rely on well-defined neuronal circuits and receptor proteins encoded by the genome, they can also be modulated by internal physiological conditions or exogenous factors

2. In the line 137-138, they found that the addition of 1% H2O2 is not sufficient to induce priming although the same concentration of H2O2 efficiently activate TrpA1+/Gr66a+ neuron. Did they try different concentrations of H_2O_2? How do they explain or speculate about this result?

As requested by this reviewer, we tested higher H_2O_2 concentrations and repeated exposures.

Our new data show that neither application of 2% H_2O (on eggs only) nor two applications (on eggs and 24-h-old larvae; 2×) were sufficient to induce priming. The new data are presented in Figure 1H and Figures S5B and S5C.

In the discussion, we added the following text.

Our results suggest that uracil acts through the ROS production system, with this step taking place in the digestive tract. Indeed, in the absence of Duox activity specifically in the gut, bacteria lose their ability to prime larvae. The observation that a similar blockage occurs upon overexpression of catalase under the *mex-GAL4* promoter supports the idea that ROS must be generated in the gut, and further suggests that hydrogen peroxide is the main ROS involved.
Importantly, we demonstrated that uracil exposure increases ROS levels in larvae, further supporting a link between Duox activation and the priming response. Although several lines of evidence indicate that ROS are required for this process, our supplementation experiments suggest that ROS alone may not be sufficient to trigger priming. Whereas dietary uracil consistently induced the phenotype, exogenous H_2O_2 failed to do so, even after testing several concentrations and exposure regimens. Because exposure to 5% H_2O_2 caused detrimental effects, we subsequently tested lower concentrations

(1%, 2%, and two successive exposures to 1% separated by 24 h), but none reproduced the effect of uracil.
One possibility would be that our supplementation protocol does not fully recapitulate the highly localized and transient production of ROS that occurs within the intestinal lumen following uracil sensing and Duox activation. Global exposure of the larvae to ROS could have detrimental effects that interfere with priming. Beyond this spatial constraint, it could be that ROS themselves may not constitute the biologically relevant signal. Instead, locally generated ROS could promote the oxidation of lipids or other biomolecules present in the gut lumen, leading to the formation of secondary metabolites such as reactive electrophilic metabolites. In vertebrates, lipid peroxidation-derived aldehydes, such as 4-hydroxynonenal (4-HNE), activate TRPA1 and contribute to physiological responses to oxidative stress [59]. More generally, TRPA1 channels are activated by a broad range of reactive electrophiles through covalent modification of conserved cysteine residues [60] and are now recognized as sensors of oxidative stress and tissue damage [61], [62]. Although this mechanism has not yet been demonstrated in the *Drosophila* intestine, it provides an attractive explanation for our observations. Uracil-induced Duox activation could generate secondary electrophilic metabolites specifically within the intestinal lumen. Such metabolites would not necessarily be produced following dietary H_2O_2 supplementation, even if ROS levels were increased, thereby explaining why exogenous H_2O_2 fails to phenocopy uracil exposure.

3. In the line 234-235: the sentence "To do so, we exposed in liquid larvae that have been raised on antibiotic-containing food to 1 mM uracil or water for 3 hr." is not clearly understandable. Perhaps the authors intended the meaning of " To do so, larvae reared on antibiotic-containing food were transferred to liquid medium and incubated with 1 mM uracil or water for 3 hours."

The text has been changed as follow.

To do so, larvae reared on antibiotic-containing food were transferred to liquid medium and incubated with 1 mM uracil or water for 3 hours.

4. How do the authors envision that larval TrpA1 activation modifies the adult gustatory circuit? What mechanisms might underlie this process? For instance, could larval TrpA1 activation induce activity-dependent synaptic or transcriptional changes during metamorphosis, thereby configuring the adult gustatory circuit to interpret PGN as an aversive stimulus? These interesting possibilities would merit discussion in the Discussion section.

In the discussion, we added the following section to discuss this matter.

A central unresolved question is how microbial information acquired during the larval stage is transmitted across metamorphosis to shape adult sensory and behavioral outputs. This transition involves extensive tissue remodeling, including the destruction and reprogramming of larval structures, cycles of dedifferentiation and redifferentiation, and profound reorganization of neuronal circuits and epigenetic landscapes. Despite this dramatic rewiring, larval microbial exposure can lead to stable alterations in adult gustatory responses, suggesting the existence of persistent or re-encoded traces of early-life experience.
One possibility is that such information is maintained at the level of defined neural circuits. In this context, the TrpA1-B+ neuron emerges as a key candidate node linking microbial sensing to downstream behavioral adaptation. However, whether this neuron acts in isolation or as part of a broader network remains unclear. Sensory responses to bacterial cues in Drosophila are typically

encoded by distributed circuits integrating gustatory, nociceptive, and neuromodulatory inputs. Mapping approaches such as GRASP or trans-Tango could therefore be used to identify synaptic partners of TrpA1-B+ cells and determine whether larval microbial experience reshapes a localized pathway or a distributed network. Such circuit-level analyses would clarify whether "priming" reflects a modification of a single sensory node or of a larger integrative circuit controlling adult aversion and feeding decisions.

Beyond circuit persistence, larval experience may also be encoded through transient functional states that are later re-established in the adult nervous system. Although metamorphosis involves the elimination or remodeling of many larval neurons, some peripheral and central gustatory neurons are known to persist or be re-specified, raising the possibility of partial continuity in sensory representations. However, given that adult proboscis responses rely on de novo sensory structures, a purely structural continuity model appears insufficient.

In parallel, more stable forms of information storage may also contribute to the persistence of larval experience across metamorphosis. Transcriptional reprogramming during development is extensive, yet certain neuronal populations retain conserved expression signatures or undergo tightly regulated fate transitions. Similarly, epigenetic mechanisms involving histone modifications and chromatin remodeling have been implicated in long-term developmental memory and could provide a substrate for encoding microbial history in specific neuronal subsets. Such mechanisms may be particularly relevant in restricted populations such as *Gr66a*- or *ppk23*-expressing neurons, which are known to participate in both larval and adult chemosensory circuits.

Finally, it is also possible that larval microbial exposure induces a form of distributed or associative-like memory that is not stored in a single neuronal substrate but instead emerges from coordinated changes across multiple levels of organization, including synaptic connectivity, transcriptional state, and epigenetic regulation.

Together, these hypotheses are not mutually exclusive. The most parsimonious model is likely one in which Duox-dependent ROS signaling initiates the formation of local electrophilic metabolites that act on sensory pathways, while parallel circuit-level and epigenetic mechanisms stabilize the resulting state across metamorphosis.

Reviewer #2:

1. Several sections of the manuscript and figure legends lack clarity. Figure panels are often insufficiently described, and key terms (e.g., ATB) should be defined upon first appearance. Each panel should be explicitly explained to allow readers to interpret the data independently of the main text.

A deep rewriting of the figure legends as well as material and methods section has been performed. An extensive new description of the procedure has been included in the legend of the figure 1 that introduces the main protocol:

Fig. 1 Larval exposure to uracil is required for adult PGN-mediated inhibition of PER.

(**A**) Live *L. brevis* are necessary and sufficient to prime the larvae. Sterilized eggs were exposed to Live or dead bacteria as well as supernatant. The resulting adults were raised on antibiotics-containing media (ATB). PER index of *w-* flies to control solutions of sucrose and sucrose + PGN from *E. coli* K12 (PGN) were obtained. For this PER datasets and the others, each fly 'able to respond' (see Material and Methods section) is attributed a 0 when it does not extend the proboscis toward the tested solution while it is attributed a 1 when it extends the proboscis toward the tested solution. Each fly represents an independent experiment and the PER index is calculated as the percentage of flies tested that

responded with a PER to the TEST stimulation and represented with the ± 95% Confidence Interval (the range of values in which the average value obtained from a complete population, and not a sample would be at 95%). For instance, if out of 30 tested animals, 21 extended their proboscis toward the tested solution, the PER index will be of 0.7. As the values obtained from one fly are categorical data with a *Yes* or *No* value, we used the Fisher exact t-test and the 95% CI to test the statistical significance of a possible difference between a test sample and the related control. This test takes into account the response of each fly and does not simply compare the distribution of several values without taking into account the size of the cohort used to generate this value. For PER assays, at least 3 independent experiments involving up to 18 flies each were performed. The results from all the flies were gathered and the total amount of animals tested is indicated in the graph. In addition, we do not show the average response from one experiment representative of the different biological replicates, but the PER index from all the tested animals in one graph. While several independent batches of flies are tested, the final PER index includes all the flies and the PER index of each independent batch is also shown as open dots in the graphs to illustrate the variability. (**B**) Uracil is necessary and sufficient to prime the larvae. Sterilized eggs were exposed to the different treatments and the resulting adults raised on antibiotics-containing media (ATB). Specifically, eggs were either mono-associated with bacterial strains, or exposed to LB media or to LB media containing uracil or cytosine. Addition of uracil or *Ecc*[15] 24h after sterilized egg deposition was tested.

2. The statement (lines 66-70) that ppk23+ neurons directly detect PGN in adults, whereas Gr66a+ neurons must be active during larval stages to establish adult PGN sensitivity, relies heavily on prior work. In the current study, the functional relationship between ppk23 and Gr66a neurons is not clearly established. Is there physiological evidence linking larval priming in Gr66a neurons to altered PGN detection in adult ppk23 neurons? Direct electrophysiological or calcium imaging data demonstrating circuit-level changes would substantially strengthen this claim.

We tested whether larval priming with uracil could modulate the responsiveness of adult *ppk23+* neurons, and we performed GCaMP calcium imaging experiments. Specifically, we compared the neuronal responses to PGN in adults originating from larvae exposed to uracil with those in adults derived from non-exposed larvae. The new data are presented in Figure S3 and the following text as has been added.

Our previous report, together with the present study, establishes a link between larval experience and the ability of adult flies to discriminate between a food mixture containing sucrose alone and one containing sucrose supplemented with PGN. We demonstrated that this discrimination relies on a subset of *ppk23+* neurons located in the adult proboscis. Exposure to PGN induced a measurable increase in intracellular calcium levels in these neurons, indicating their activation by bacterial cues. In contrast, *ppk23+* neurons from adults derived from larvae not exposed to bacteria failed to respond to PGN stimulation [37].
To investigate whether larval priming with uracil could modulate the responsiveness of adult *ppk23+* neurons, we performed GCaMP calcium imaging experiments. Specifically, we compared the neuronal responses to PGN in adults originating from larvae exposed to uracil with those in adults derived from non-exposed larvae. This approach allowed us to examine, at the cellular level, how early-life exposure to microbial-associated signals influences the development of sensory discrimination mechanisms in adulthood. While *ppk23+* proboscis's neurons of adults from larvae exposed or not to uracil responded to salt (positive controls), only the adults originating from uracil-exposed larvae responded to PGN (Figure S3)

3. The manuscript suggests that transient activation of TO (TrpA1+) neurons during larval stages induces adult PGN aversion. Is short-term activation (3-12 hr) by quinine or H_2O_2 sufficient to establish this long-term behavioral change? Is H_2O_2 specifically required, or could quinine-induced activation also produce priming independently of ROS? These experiments would clarify whether ROS signaling is mechanistically necessary or whether generic TRPA1 activation is sufficient.

We tested higher H_2O_2 concentrations and repeated exposure and added the following text as well as a figure.

However, using either the monoassociation protocol (Figure S1B) or axenic media (ATB), neither a single application of 1% H_2O_2 (on eggs only) nor two applications (on eggs and 24-h-old larvae; 2×), nor 2% H_2O_2, were sufficient to induce priming (Figure 1H and Figures S5B and S5C).

We tested quinine, added the following text as well as a figure.

Having demonstrated that this larval neuron was required for priming and can be activated by quinine, we tested whether larval exposure to quinine using the monoassociation protocol was sufficient to prime larvae, as previously shown for uracil, and found that it was not (Figure S10G).

5. The proposed mechanism hinges on uracil-induced ROS production. Direct quantification of H_2O_2 or total ROS levels in uracil-exposed larval media is essential, along with appropriate negative controls (e.g., Duox mutants, antioxidant treatment). Without biochemical validation, the ROS-dependent priming model remains inferential.

As requested by this reviewer, we tested the presence of H_2O_2 and ROS and added the new data in Figure 6. The new results were described by the following text.

Section: **The production of reactive oxygen species by enterocytes is necessary for larval priming**
Based on the genetic data and the identified critical period, we tested whether ROS, and H_2O_2 in particular, could be detected in 24-h-old larvae following exposure of sterilized eggs to uracil. To confirm that the measured signal corresponded to H_2O_2, we either treated uracil-exposed samples with catalase or genetically overexpressed catalase (an enzyme that degrades hydrogen peroxide) in enterocytes (*mex-GAL4/UAS-Catalase*). Our results demonstrate that H_2O_2 levels are increased following uracil exposure (Figure 1F).

Section: **Uracil induces a Duox-dependent signal activating *TrpA1-B+* neurons in naïve larvae**
To determine whether ROS is produced by larvae under these conditions and to characterize its time course, we measured ROS levels over time using a chemiluminescent probe in wells containing exposed larvae. Larvae exposed to buffer exhibited a basal level of ROS that remained stable over the course of the experiment (Figure 6D). This signal was abolished upon addition of vitamin C (VitC). In contrast, addition of uracil triggered an increase in ROS compared to buffer alone; this signal then gradually decreased over time. At 3 hours, ROS levels following uracil exposure remained higher than in the buffer condition.

6. TRPA1 expression in bitter-sensing GRNs, including Gr66a neurons, has been well established. Prior work demonstrating Gr66a-TrpA1 coexpression (e.g., studies from Craig Montell's laboratory) should be acknowledged and appropriately integrated into the part to contextualize the findings.

We added new original references for TrpA1 in drosophila and the text as well as a reference including pioneering ones from Craig Montell's laboratory:

Drosophila TrpA1, which shares conserved sensory functions with its mammalian ortholog and is activated by heat, ROS, UV light, and irritant chemicals (Figure 2A), is alternatively spliced to produce five isoforms (Figure 2B) [46], [47], [48], [49], [50], [51].

This suggests that *TrpA1-B-GAL4* and *Gr66a-GAL4* are co-expressed in one or more cells in which *TrpA1* expression would be required for priming. *TprA1* expression in bitter-sensing GRNs, including *Gr66a+* neurons, has been well established in adults [49], [54], [55].

[49] S. H. Kim, Y. Lee, B. Akitake, O. M. Woodward, W. B. Guggino, et C. Montell, « Drosophila TRPA1 channel mediates chemical avoidance in gustatory receptor neurons », *Proc Natl Acad Sci U S A*, vol. 107, n° 18, p. 8440-8445, mai 2010, doi: 10.1073/pnas.1001425107.
[50] M. Rosenzweig, K. M. Brennan, T. D. Tayler, P. O. Phelps, A. Patapoutian, et P. A. Garrity, « The Drosophila ortholog of vertebrate TRPA1 regulates thermotaxis », *Genes Dev*, vol. 19, n° 4, p. 419-424, févr. 2005, doi: 10.1101/gad.1278205.
[51] C. Montell, « The TRP superfamily of cation channels », *Sci STKE*, vol. 2005, n° 272, p. re3, févr. 2005, doi: 10.1126/stke.2722005re3.
[54] A. R. Guntur *et al.*, « H2O2-Sensitive Isoforms of Drosophila melanogaster TRPA1 Act in Bitter-Sensing Gustatory Neurons to Promote Avoidance of UV During Egg-Laying », *Genetics*, vol. 205, n° 2, p. 749-759, févr. 2017, doi: 10.1534/genetics.116.195172.
[55] A. Soldano *et al.*, « Gustatory-mediated avoidance of bacterial lipopolysaccharides via TRPA1 activation in Drosophila », *Elife*, vol. 5, p. e13133, juin 2016, doi: 10.7554/eLife.13133.

7. Figure 6 is difficult to interpret. What is the precise stimulus applied before and after conditioning? Is the conditioned medium itself the stimulus?

The text has been changed as follow.

To do so, larvae reared on antibiotic-containing food were transferred to liquid medium and incubated with 1 mM uracil or water for 3 hours. The resulting conditioned medium was assayed for calcium responses in naïve, non–uracil-exposed animals (Figure 6A). Strikingly, while neither uracil alone nor water from larvae not exposed to uracil induced any response, the medium conditioned by uracil-treated larvae triggered a calcium rise in the anteriormost TrpA1-B+ neuron (Figure 6B, 6C and S11). Importantly, when Duox expression was specifically reduced in enterocytes of the uracil-treated larvae (mex-Gal4/UAS-Duox_IR), the conditioned medium no longer activated the TrpA1-B+ neuron (Figure 6B-C).

8. Representative calcium traces should be shown alongside clear indication of stimulus onset and response timing. Without this information, it is difficult to evaluate the physiological relevance of the results.

We added calcium traces as a new Figure S11.

Strikingly, while neither uracil alone nor water from larvae not exposed to uracil induced any response, the medium conditioned by uracil-treated larvae triggered a calcium rise in the anteriormost TrpA1-B+ neuron (Figure 6B, 6C and S11).

Fig. S11 Time course response of the *TrpA1-B+* neuron responding to medium conditioned by larvae exposed to uracil

Averaged time course of the GCaMP intensity variations (ΔF/F0 %) for *TrpA1-B+* neurons (*TrpA1-B-GAL4/UAS-GCaMP7s*) in response to either the chemicals used in the conditioned media assay (uracil; n=7) or the medium in which the larvae were incubated in (media form *w*$^-$ larvae+water (n=8) or media form *w*$^-$ larvae+uracil 1mM (n=6). The addition of the chemical or the conditioned media at a specific time is indicated by the arrow.

9. Most experiments appear to rely on only three biological replicates. Given the complexity of developmental priming and behavioral assays, increased biological replication is necessary to ensure robustness and reproducibility.

We respectfully disagree that the number of replicates is insufficient. In the PER assay, each fly is tested individually and constitutes a single experimental observation. For each condition, at least three biologically independent experiments were performed, with around 10 flies analyzed individually in each experiment. Thus, the dataset comprises at least 30 individual observations collected across three independent biological replicates.

Importantly, each fly was assayed individually using its own internal controls, and the response was scored at the level of the individual animal. Therefore, statistical analyses were performed on individual observations rather than on experiment-level averages. The response was scored as a binary outcome at the level of the individual animal. Therefore, statistical analyses were performed on individual observations using Fisher's exact test, which is specifically designed for categorical data and makes full use of all observed responses. Furthermore, the observed effect was reproducibly detected across independent experimental replicates, indicating that the conclusions are not driven by a single experiment but are consistently observed in separate biological replicates. If we only consider conditions demonstrating 'priming', we observed it with more than 600 individual flies and more than 10 different contexts (genotypes, raising conditions).

We added the following new text to explain and clarify the statistics in the Material and Methods section.

M&M section: **Statistics**

For PER datasets, each fly 'able to respond' is attributed a 0 when it does not extend the proboscis toward the tested solution while it is attributed a 1 when it extends the proboscis toward the tested solution. As the values obtained from one fly are categorical data with a *Yes* or *No* value, we used the Fisher exact t-test and the 95% CI to test the statistical significance of a possible difference between a test sample and the related control.

This test takes into account the response of each fly and does not simply compare the distribution of several values without taking into account the size of the cohort used to generate this value. For PER assays, at least 3 independent experiments involving up to 18 flies each were performed. The results

from all the flies were gathered and the total amount of animals tested is indicated in the graph. In addition, we do not show the average response from one experiment representative of the different biological replicates, but the PER index from all the tested animals in one graph. Each fly represents an independent experiment and the PER index is calculated as the percentage of flies tested that responded with a PER to the TEST stimulation and represented with the ± 95% CI. For instance, if out of 30 tested animals, 21 extended their proboscis toward the tested solution, the PER index will be of 0.7. Several independent batches of flies are tested, the final PER index includes all the flies and the PER index of each independent batch is also shown as open dots in the graphs to illustrate the variability.

10. Is the priming effect specific to uracil, or could other pyrimidines (e.g., thymine) produce similar effects? Testing structurally related metabolites would clarify whether the phenomenon reflects specific microbial signaling or general nucleobase exposure.

As requested, we tested other pyrimidines. New data were incorporated in existing figure 1 as well as new figure S2 and the text was modified as follow.

Loss of priming in uracil-release mutants and sufficiency of exogenous uracil implicate uracil as a key determinant under our conditions. To delineate whether uracil-related molecules could prime larvae, we used the same protocol as the one involving uracil to expose sterilized eggs to cytosine or thymine. None of these molecules were able to phenocopy the priming effect of uracil (Figure 1B and S2).

Reviewer #3:

1.While Duox-dependent ROS production is shown to be required, the study does not directly quantify ROS levels in the larval gut. Therefore, the exact identity of the signaling molecule remains unclear. Since the addition of hydrogen peroxide did not fully rescue priming, additional factors need to be considered which might confer priming in adult flies.
Identifying the biochemical identity of this signal could further pinpoint the exact mechanism.

As requested, we tested the presence of H_2O_2 and ROS and added the following text as well as a figure

Section: **The production of reactive oxygen species by enterocytes is necessary for larval priming**
Based on the genetic data and the identified critical period, we tested whether ROS, and H_2O_2 in particular, could be detected in 24-h-old larvae following exposure of sterilized eggs to uracil. To confirm that the measured signal corresponded to H_2O_2, we either treated uracil-exposed samples with catalase or genetically overexpressed catalase (an enzyme that degrades hydrogen peroxide) in enterocytes (*mex-GAL4/UAS-Catalase*). Our results demonstrate that H_2O_2 levels are increased following uracil exposure (Figure 1F).

Section: **Uracil induces a Duox-dependent signal activating *TrpA1-B+* neurons in naïve larvae**
To determine whether ROS is produced by larvae under these conditions and to characterize its time course, we measured ROS levels over time using a chemiluminescent probe in wells containing exposed larvae. Larvae exposed to buffer exhibited a basal level of ROS that remained stable over the course of the experiment (Figure 6D). This signal was abolished upon addition of vitamin C (VitC). In contrast, addition of uracil triggered an increase in ROS compared to buffer alone; this signal then gradually decreased over time. At 3 hours, ROS levels following uracil exposure remained higher than in the buffer condition.

2-The study does not address how activation of the identified larval neurons results in lasting behavioral changes in the adult. While the introduction briefly mentions how animals perceive the environment through changes in neuronal circuits, the cellular basis of this priming remains unclear. For example, it is not known whether priming induces structural changes in sensory circuits or long-term transcriptional changes that are maintained through adulthood.
I feel this point is rather critical since many of the neural circuits from the larva are substantially reorganized during metamorphosis or even lost.

Although the point raised by the reviewer is important, we believe that it is beyond the scope of the manuscript and will require extensive work to figure out the mechanisms involved. We added the following section to the Discussion paragraph to propose some putative mechanisms.

A central unresolved question is how microbial information acquired during the larval stage is transmitted across metamorphosis to shape adult sensory and behavioral outputs. This transition involves extensive tissue remodeling, including the destruction and reprogramming of larval structures, cycles of dedifferentiation and redifferentiation, and profound reorganization of neuronal circuits and epigenetic landscapes. Despite this dramatic rewiring, larval microbial exposure can lead to stable alterations in adult gustatory responses, suggesting the existence of persistent or re-encoded traces of early-life experience.
One possibility is that such information is maintained at the level of defined neural circuits. In this context, the TrpA1-B+ neuron emerges as a key candidate node linking microbial sensing to downstream behavioral adaptation. However, whether this neuron acts in isolation or as part of a broader network remains unclear. Sensory responses to bacterial cues in Drosophila are typically encoded by distributed circuits integrating gustatory, nociceptive, and neuromodulatory inputs. Mapping approaches such as GRASP or trans-Tango could therefore be used to identify synaptic partners of TrpA1-B+ cells and determine whether larval microbial experience reshapes a localized pathway or a distributed network. Such circuit-level analyses would clarify whether "priming" reflects a modification of a single sensory node or of a larger integrative circuit controlling adult aversion and feeding decisions.
Beyond circuit persistence, larval experience may also be encoded through transient functional states that are later re-established in the adult nervous system. Although metamorphosis involves the elimination or remodeling of many larval neurons, some peripheral and central gustatory neurons are known to persist or be re-specified, raising the possibility of partial continuity in sensory representations. However, given that adult proboscis responses rely on de novo sensory structures, a purely structural continuity model appears insufficient.
In parallel, more stable forms of information storage may also contribute to the persistence of larval experience across metamorphosis. Transcriptional reprogramming during development is extensive, yet certain neuronal populations retain conserved expression signatures or undergo tightly regulated fate transitions. Similarly, epigenetic mechanisms involving histone modifications and chromatin remodeling have been implicated in long-term developmental memory and could provide a substrate for encoding microbial history in specific neuronal subsets. Such mechanisms may be particularly relevant in restricted populations such as *Gr66a*- or *ppk23*-expressing neurons, which are known to participate in both larval and adult chemosensory circuits.
Finally, it is also possible that larval microbial exposure induces a form of distributed or associative-like memory that is not stored in a single neuronal substrate but instead emerges from coordinated changes across multiple levels of organization, including synaptic connectivity, transcriptional state, and epigenetic regulation.

Together, these hypotheses are not mutually exclusive. The most parsimonious model is likely one in which Duox-dependent ROS signaling initiates the formation of local electrophilic metabolites that act on sensory pathways, while parallel circuit-level and epigenetic mechanisms stabilize the resulting state across metamorphosis.

3. Additionally, whether a critical window exists during larval development which makes an adult fly more sensitive to being primed to PGN is not fully shown.
Considering this would make the mechanism underlying long-term behavioral changes clearer.

We tested the early critical window and added the following text as well as new data in the existing figure:

In the monoassociation protocol, bacteria or chemical compounds are deposited onto sterilized eggs, and the resulting adults are subsequently tested for PER. In our previous study investigating the PER response to a sucrose + PGN mixture and the requirement for larval exposure to bacteria, we identified a critical developmental window between oviposition and 48 h post-oviposition [37]. We therefore asked whether the effect of uracil depended on the same critical period. To address this question, we exposed 24h-old larvae derived from sterilized eggs to uracil rather than exposing the eggs themselves, and subsequently assayed PER in the resulting adults. This treatment failed to prime the larvae (Figure 1B). We obtained the same results with 24 h-old larvae derived from sterilized eggs and exposed to *Ecc*[15]. These results confirm the existence of a critical developmental window and further narrow it to a period occurring before 24 h after larval hatching.

4. Since some of the experiments should uniquely address larval priming, but the Gal4 lines are also active in the adult, the respective expression in the adult might also be relevant; plus, conclusive larval-specific expression vs adult-specific expression (e.g. Gal80ts) would clarify the temporal roles; as far as I can see this has only been done for Duox?

This key experiment requested by the reviewer is present in the original Figure 4B. Using the $Gal80^{ts}$ /Gal 4 combination, we demonstrate that the inactivation of *TrpA1* (*UAS-TrpA1_IR*) in *TrpA1B+* cells (*TrpA1-B-GAL4*) impairs the priming only when *TrpA1* is silenced during the larval stage and not during the adult stage.

5. Minor improvements:
-The authors refer to hatching as "birth" (line 80; and SFig1), please correct this.

The text has been changed. larva to give rise to adults

- grammatical error in line 17- "Animals perceive their environment through their sensory systems why rely on neuronal circuits and proteins», «why rely" is grammatically incorrect in this context, which can be rephrased with "while relying on neuronal circuits and proteins."

The text has been changed.

Animals perceive their immediate environment through their sensory systems. While these systems rely on well-defined neuronal circuits and receptor proteins encoded by the genome, they can also be modulated by internal physiological conditions or exogenous factors.
