# Supplementary material for: Uracil-driven ROS signaling and larval TrpA1-B neuron activation are required for priming the adult Drosophila gustatory response to bacteria
Source: PLoS Biol. 2026 Oct 5;24(10):e3004042. doi: 10.1371/journal.pbio.3004042 (PMC13657326; doi:10.1371/journal.pbio.3004042)
Supplement: Supplementary file 2 [file pbio.3004042.s014.pdf]

! "#$%&' ()%*+(,(-.%"/+0($12%! "#$%$"3+" 4 "$12%
%
&,"#1"%5+6/%07"%8(+6090(
