# Supplementary material for: Uracil-driven ROS signaling and larval TrpA1-B neuron activation are required for priming the adult Drosophila gustatory response to bacteria
Source: PLoS Biol. 2026 Oct 5;24(10):e3004042. doi: 10.1371/journal.pbio.3004042 (PMC13657326; doi:10.1371/journal.pbio.3004042)
Supplement: Supplementary file 3 [file pbio.3004042.s015.docx]

Der Editors,

Thank you sending us your editorial decision together with the reviewers ‘appreciation. Here is our response to your and the reviewers’ suggestions.

Thank you for your patience while we considered your revised manuscript "Uracil-Driven ROS signals activate larval TrpA1-B neurons to prime the Drosophila adult gustatory response to bacterial signal" for publication as a Research Article at PLOS Biology. This revised version of your manuscript has been evaluated by the PLOS Biology editors, the Academic Editor, and the original reviewers.

Based on the reviews, we are likely to accept this manuscript for publication, provided you satisfactorily address the remaining points raised by Reviewer 2. Please also make sure to address the following data and other policy-related requests.

IMPORTANT: Please ensure that your next revision addresses all of the following editorial points:

---------------------------

**Title:

We would suggest to tweak the title slightly in accordance with the remaining points from Reviewer 2. Specifically, we suggest to change 'ROS signals' to 'ROS signaling' to account for the remaining uncertainty about the direct or indirect role of ROS, and to use wording in the title that the identified signaling elements are necessary but not necessarily sufficient on their own. Is this following alternative version acceptable to you?

"Uracil-driven ROS signaling and larval TrpA1-B neuron activation are required for priming the adult Drosophila gustatory response to bacteria"

Yes, we agree. This has been done

**Financial disclosure statement:

The Financial disclosure statement should include URLs for the funding agencies. It should also directly answer the following question: "Did the sponsors or funders play any role in the study design, data collection and analysis, decision to publish, or preparation of the manuscript?"

This has been done 

**Data:


Please change the Data Availability Statement to match our guidelines in the submission form, e.g. following this format:

"The minimal data set [and accompanying code, where generated] is available at [repository name and/or 'in the Supporting Information'] via [DOI hyperlink and/or S1 Data]."

Since you have included the data both on Figshare and in the uploaded source data file, we would suggest that you mention both in the Data Availability Statement.

If you choose to keep the uploaded data file, please re-name it as 'S1 Data' ('S1_Data.xlsx') and include its title/legend in the manuscript file, after those of the supplementary figures.

This has been done 

In addition, please cite the location of the data clearly in all relevant main and supplementary Figure legends, e.g. “The data underlying this Figure can be found in S1 Data” or “The data underlying this Figure can be found in [https://doi.org/10.5281/zenodo.XXXXX”](https://doi.org/10.5281/zenodo.XXXXX%E2%80%9D).

This has been done 

 **Code availability:
 
Per journal policy, if you have generated any custom code or scripts during the course of this investigation, we require that you make it available without restrictions. Please ensure that the code is sufficiently well documented and reusable, and that your Data Statement in the Editorial Manager submission system accurately describes where your code can be found. Please also ensure that you choose a license for your code and include a Readme file.
 
Please note that we cannot accept sole deposition of code in GitHub, as this could be changed after publication. However, you can archive this version of your publicly available GitHub code to Zenodo. Once you do this, it will generate a DOI number, which you will need to provide in the Data Accessibility Statement (you are welcome to also provide the GitHub access information). See the process for doing this here: <https://docs.github.com/en/repositories/archiving-a-github-repository/referencing-and-citing-content>


---------------------------
As you address these items, please take this last chance to review your reference list to ensure that it is complete and correct. If you have cited papers that have been retracted, please include the rationale for doing so in the manuscript text, or remove these references and replace them with relevant current references. Any changes to the reference list should be mentioned in the cover letter that accompanies your revised manuscript.

In addition to these revisions, you may need to complete some formatting changes, which you will receive in a follow up email. A member of our team will be in touch with a set of requests shortly. If you do not receive a separate email within a few days, please assume that checks have been completed, and no additional changes are required.

We expect to receive your revised manuscript within two weeks.

To submit your revision, please go to <https://www.editorialmanager.com/pbiology/> and log in as an Author. Click the link labelled 'Submissions Needing Revision' to find your submission record. Your revised submission must include the following:
-  a cover letter that should detail your responses to any editorial requests, if applicable, and whether changes have been made to the reference list
-  a Response to Reviewers file that provides a detailed response to the reviewers' comments (if applicable, if not applicable please do not delete your existing 'Response to Reviewers' file.)
-  a track-changes file indicating any changes that you have made to the manuscript.

NOTE: If Supporting Information files are included with your article, note that these are not copyedited and will be published as they are submitted. Please ensure that these files are legible and of high quality (at least 300 dpi) in an easily accessible file format. For this reason, please be aware that any references listed in an SI file will not be indexed. For more information, see our Supporting Information guidelines:
<https://journals.plos.org/plosbiology/s/supporting-information> 

*Published Peer Review History*
Please note that you may have the opportunity to make the peer review history publicly available. The record will include editor decision letters (with reviews) and your responses to reviewer comments. If eligible, we will contact you to opt in or out. Please see here for more details:
<https://plos.org/published-peer-review-history/>

*Press*
Should you, your institution's press office or the journal office choose to press release your paper, please ensure you have opted out of Early Article Posting on the submission form. We ask that you notify us as soon as possible if you or your institution is planning to press release the article.

*Protocols deposition*
To enhance the reproducibility of your results, we recommend that if applicable you deposit your laboratory protocols in protocols.io, where a protocol can be assigned its own identifier (DOI) such that it can be cited independently in the future. Additionally, PLOS ONE offers an option for publishing peer-reviewed Lab Protocol articles, which describe protocols hosted on protocols.io. Read more information on sharing protocols at <https://plos.org/protocols?utm_medium=editorial-email&utm_source=authorletters&utm_campaign=protocols>


Please do not hesitate to contact me should you have any questions.

Sincerely,

Taylor

Taylor Hart, PhD,
Associate Editor
thart@plos.org
PLOS Biology


Reviewer remarks:

Reviewer #1: I thank the authors for their careful and thorough revision of the manuscript. They have addressed all of my previous comments satisfactorily. I believe the revised manuscript is significantly improved and is suitable for publication.

Reviewer #2: The authors propose that exposure of Drosophila larvae to bacterial uracil activates Duox-dependent oxidative signaling in the gut, which is then detected by a TrpA1-B⁺/Gr66a⁺ neuron in the larval terminal organ, ultimately modifying the adult gustatory response to bacterial peptidoglycan. This is an interesting concept because it links an early microbial experience to long-lasting changes in adult sensory behavior.
Overall, the revision has substantially improved the manuscript. The authors have addressed many of the previous concerns by (1) expanding the figure legends and defining ATB in the main protocol; (2) adding calcium-imaging data from adult ppk23⁺/Gr66a⁻ neurons following larval uracil exposure; (3) testing higher concentrations and repeated exposure to H₂O₂, as well as quinine exposure; (4) adding ROS/H₂O₂ measurements together with a conditioned-medium experiment; (5) incorporating previous literature regarding TrpA1 expression in bitter-sensing neurons; and (6) providing an averaged calcium-response time course while testing cytosine and thymine as related pyrimidines.
Overall, I appreciate the authors' efforts, and several aspects of the manuscript are now considerably stronger. Nevertheless, I believe a few minor issues still deserve attention before publication.
1. The revisions to Figure 1 and the definition of ATB have improved clarity. However, the Figure 1 legend has become unnecessarily long and now includes detailed statistical descriptions that would be more appropriate in the Materials and Methods section.

This has been modified.

In addition, two statements should be corrected. The phrase "each fly represents an independent experiment" is inaccurate because each fly is an independent observation rather than an independent experiment.

This has been corrected.

Likewise, "Fisher exact t-test" is not a valid statistical test and should be corrected to "Fisher's exact test."`

This has been corrected.

2. Although the measured ROS increase was not directly examined in Duox-knockdown larvae and the precise downstream signaling molecule remains unidentified, I do not consider these limitations sufficient to preclude publication. The current data support the involvement of oxidative signaling, but the manuscript should maintain appropriately cautious language and avoid presenting H₂O₂ as the conclusively identified instructive signal or sole mediator of priming. Increased ROS or hydrogen peroxide is not equivalent to identifying the final biochemical signal responsible for the developmental effect.

This has been done with changing the titles and highlights and in the discussion, we do not present H₂O₂ as the identified instructive signal or sole mediator of priming.

3. My remaining concern relates to the strength of the mechanistic conclusions. The title states that "Uracil-Driven ROS signals activate larval TrpA1-B neurons," whereas the Discussion also proposes that ROS may instead generate secondary electrophilic metabolites that ultimately activate TrpA1. These are distinct mechanistic possibilities and should not be presented interchangeably.
More importantly, the newly added experiments show that activating terminal organ (TrpA1⁺) neurons during larval stages with either H₂O₂ or quinine failed to induce adult peptidoglycan aversion, despite confirming neuronal activation. This suggests that activation of these neurons alone is insufficient to account for uracil-dependent priming. Accordingly, the manuscript should either directly test neuronal sufficiency using a selective activation strategy (e.g., optogenetic, thermogenetic, or chemogenetic activation) or revise the title, Highlights, and conclusions to state more explicitly that TrpA1-B neuron activity is necessary but has not been shown to be sufficient, while briefly discussing additional uracil-specific signals that may be required.

We have changed the title to “Uracil-driven ROS signaling and larval TrpA1-B neuron activation are required for priming the adult Drosophila gustatory response to bacteria”

More broadly, the current data do not demonstrate a direct synaptic connection between the larval TrpA1-B-positive neuron and the adult ppk23-positive neuronal population, nor do they distinguish among alternative mechanisms such as altered developmental connectivity, receptor expression, neuronal excitability, or broader developmental programming. Therefore, describing the findings as a complete circuit-level mechanism appears somewhat stronger than the available evidence supports.

We do not believe that we present these findings as a “complete circuit-level mechanism.” Rather, we present data showing that modulation of neuronal activity during the larval stage has consequences for the adult sensory system. We also explicitly acknowledge in the Discussion that the underlying mechanisms remain unknown and devote an entire paragraph to proposing potential mechanisms that could be tested in future studies.

Finally, the adult ppk23 calcium-imaging data should be described as evidence of altered adult neuronal responsiveness following larval experience.

This has been added

There is a typographical error in which TrpA1 is incorrectly written as TprA1.

This has been corrected


Reviewer #3 [Simon Sprecher]: The authors have addressed all points raised, I feel it's an important and interesting contribution.

Dear Royet,

Thank you for your patience while we performed technical checks on your submission. Please see below for a list of requirements for you to address while conducting your revisions.

Journal Requirements:

1) Please amend your detailed Financial Disclosure statement. This is published with the article, therefore should be completed in full sentences and contain the exact wording you wish to be published.

This has been done

i). State what role the funders took in the study. If the funders had no role in your study, please state: “The funders had no role in study design, data collection and analysis, decision to publish, or preparation of the manuscript.”

This has been done

ii). If any authors received a salary from any of your funders, please state which authors and which funders.

This has been done

2) Please ensure that the funders and grant numbers match between the Financial Disclosure field and the Funding Information tab in your submission form. Note that the funders must be provided in the same order in both places as well. Currently, " Y.G. Research in Y.G.’s laboratory is supported by the CNRS, the “Université de Bourgogne Europe”, the Conseil Régional Bourgogne Franche-Comté (PARI grant), the FEDER (European Funding for Regional Economical Development)" are missing from the Funding Information

This has been done

3) We ask that a manuscript source file is provided at Revision. Please upload your manuscript file as a .doc, .docx, .rtf or .tex.

NOTE: If manuscript is a LaTeX submission, Please ensure that you provide a single, cohesive .tex source file for your LaTeX revision. You may upload this file as the item type 'LaTeX Source File.' Please also ensure that you are making any formatting changes to both your .tex file and the PDF of your manuscript. If you have any questions, please contact customercare@plos.org. You can find our LaTeX guidelines here:
<https://journals.plos.org/plosbiology/s/latex>  

4) Please provide separate figure files in .tif or .eps format. For more information about figure files please see our guidelines:

<https://journals.plos.org/plosbiology/s/figures> 
<https://journals.plos.org/plosbiology/s/figures#loc-file-requirements> 

5) We have noticed that you have uploaded "Source data_PGN_MAJ 25-06.xlsx" file as supporting information but you have not included it in the list of legends.  Please add a citation of this SI data file into the list of legends of your supporting information files.

This has been done

6) Please refer to the following article for PLOS Biology's best-practice recommendations for data presentation: <https://journals.plos.org/plosbiology/article?id=10.1371/journal.pbio.1002128>.
Guidelines for statistical reporting, if relevant, may be found here: <https://journals.plos.org/plosbiology/s/best-practices-in-research-reporting#loc-statistical-reporting>.

7) Some material included in your submission may be copyrighted. According to PLOS’s copyright policy, authors who use figures or other material (e.g., graphics, clipart, maps) from another author or copyright holder must demonstrate or obtain permission to publish this material under the Creative Commons Attribution 4.0 International (CC BY 4.0) License used by PLOS journals. Please closely review the details of PLOS’s copyright requirements here: PLOS Licenses and Copyright. If you need to request permissions from a copyright holder, you may use PLOS's Copyright Content Permission form.

Please respond directly to this email and provide any known details concerning your material's license terms and permissions required for reuse, even if you have not yet obtained copyright permissions or are unsure of your material's copyright compatibility. Once you have responded and addressed all other outstanding technical requirements, you may resubmit your manuscript within Editorial Manager.

Potential Copyright Issues:

i) [Figure 2,3,5A,5E,6A,S1,S4,S6,S7,S8,S9,S10 Figure]: Please confirm whether you drew the images / clip-art within the figure panels by hand. If you did not draw the images, please provide (a) a link to the source of the images or icons and their license / terms of use; or (b) written permission from the copyright holder to publish the images or icons under our CC BY 4.0 license. Alternatively, you may replace the images with open source alternatives. See these open source resources you may use to replace images / clip-art:
- [https://commons.wikimedia.org](https://commons.wikimedia.org/)
- <https://openclipart.org/>

ii) [Figure 7,S4,S5,S8]: Please confirm (a) that you are the photographer; or (b) provide written permission from the photographer to publish the photo(s) under our CC BY 4.0 license.

Yes, the authors are the photographers.

If you have any questions or require assistance, please don't hesitate to get in touch via return email. To help us minimize inbox volumes, we kindly ask that you not reply to this email simply to confirm you have resubmitted your revisions.

Kind regards,

Nithya Venkatesan
Publications Assistant
PLOS Biology
plosbiology@plos.org
