# Supplementary material for: Uracil-driven ROS signaling and larval TrpA1-B neuron activation are required for priming the adult Drosophila gustatory response to bacteria
Source: PLoS Biol. 2026 Oct 5;24(10):e3004042. doi: 10.1371/journal.pbio.3004042 (PMC13657326; doi:10.1371/journal.pbio.3004042)
Supplement: Peer review history

# Author comment: Author response to Decision Letter 0

**DOI:** 10.1371/journal.pbio.3004042.r001

2 Jul 2026

Attachment Submitted filename: RM et al, Response to reviewer July 2.pdf

# Editor report: Decision Letter 0

**DOI:** 10.1371/journal.pbio.3004042.r002

**Authors:** Taylor Hart, PhD

16 Jul 2026

Dear Dr Royet,

Thank you for submitting the revised version of your manuscript entitled "Uracil-Driven ROS signals activate larval TrpA1-B neurons to prime the Drosophila adult gustatory response to bacterial signal" for further consideration as a Research Article by PLOS Biology.

Your manuscript has now been evaluated by the PLOS Biology editorial staff, and I am writing to let you know that we would like to send your submission out for further external peer review, aiming to recruit the same reviewers who advised on the earlier version of the submission.

As this is treated in the Editorial Manager system as a new submission, we ask that you add some required metadata before we can send this out to the reviewers. To this end, please login to Editorial Manager where you will find the paper in the 'Submissions Needing Revisions' folder on your homepage. Please click 'Revise Submission' from the Action Links and complete all additional questions in the submission questionnaire.

Once your full submission is complete, your paper will undergo a series of checks in preparation for peer review. After your manuscript has passed the checks it will be sent out for review. To provide the metadata for your submission, please Login to Editorial Manager (https://www.editorialmanager.com/pbiology) within two working days, i.e. by Jul 18 2026 11:59PM.

During the process of completing your manuscript submission, you will be invited to opt-in to posting your pre-review manuscript as a bioRxiv preprint. Visit http://journals.plos.org/plosbiology/s/preprints for full details. If you consent to posting your current manuscript as a preprint, please upload a single Preprint PDF.

Feel free to email us at plosbiology@plos.org if you have any queries relating to your submission.

Kind regards,

Taylor

Taylor Hart, PhD,

Associate Editor

PLOS Biology

thart@plos.org

# Author comment: Author response to Decision Letter 1

**DOI:** 10.1371/journal.pbio.3004042.r003

23 Jul 2026

Attachment Submitted filename: RM_et_al,_Response__to_reviewer_July_2_auresp_1.pdf

# Peer review: Decision Letter 1

**DOI:** 10.1371/journal.pbio.3004042.r004

**Authors:** Taylor Hart, PhD

1 Sep 2026

Dear Dr Royet,

Thank you for your patience while we considered your revised manuscript "Uracil-Driven ROS signals activate larval TrpA1-B neurons to prime the Drosophila adult gustatory response to bacterial signal" for publication as a Research Article at PLOS Biology. This revised version of your manuscript has been evaluated by the PLOS Biology editors, the Academic Editor, and the original reviewers.

Based on the reviews, we are likely to accept this manuscript for publication, provided you satisfactorily address the remaining points raised by Reviewer 2. Please also make sure to address the following data and other policy-related requests.

IMPORTANT: Please ensure that your next revision addresses all of the following editorial points:

---------------------------

**Title:

We would suggest to tweak the title slightly in accordance with the remaining points from Reviewer 2. Specifically, we suggest to change 'ROS signals' to 'ROS signaling' to account for the remaining uncertainty about the direct or indirect role of ROS, and to use wording in the title that the identified signaling elements are necessary but not necessarily sufficient on their own. Is this following alternative version acceptable to you?

"Uracil-driven ROS signaling and larval TrpA1-B neuron activation are required for priming the adult Drosophila gustatory response to bacteria"

**Financial disclosure statement:

The Financial disclosure statement should include URLs for the funding agencies. It should also directly answer the following question: "Did the sponsors or funders play any role in the study design, data collection and analysis, decision to publish, or preparation of the manuscript?"

**Data:

Please change the Data Availability Statement to match our guidelines in the submission form, e.g. following this format:

"The minimal data set [and accompanying code, where generated] is available at [repository name and/or 'in the Supporting Information'] via [DOI hyperlink and/or S1 Data]."

Since you have included the data both on Figshare and in the uploaded source data file, we would suggest that you mention both in the Data Availability Statement.

If you choose to keep the uploaded data file, please re-name it as 'S1 Data' ('S1_Data.xlsx') and include its title/legend in the manuscript file, after those of the supplementary figures.

In addition, please cite the location of the data clearly in all relevant main and supplementary Figure legends, e.g. “The data underlying this Figure can be found in S1 Data” or “The data underlying this Figure can be found in https://doi.org/10.5281/zenodo.XXXXX”.

**Code availability:

Per journal policy, if you have generated any custom code or scripts during the course of this investigation, we require that you make it available without restrictions. Please ensure that the code is sufficiently well documented and reusable, and that your Data Statement in the Editorial Manager submission system accurately describes where your code can be found. Please also ensure that you choose a license for your code and include a Readme file.

Please note that we cannot accept sole deposition of code in GitHub, as this could be changed after publication. However, you can archive this version of your publicly available GitHub code to Zenodo. Once you do this, it will generate a DOI number, which you will need to provide in the Data Accessibility Statement (you are welcome to also provide the GitHub access information). See the process for doing this here: https://docs.github.com/en/repositories/archiving-a-github-repository/referencing-and-citing-content

---------------------------

As you address these items, please take this last chance to review your reference list to ensure that it is complete and correct. If you have cited papers that have been retracted, please include the rationale for doing so in the manuscript text, or remove these references and replace them with relevant current references. Any changes to the reference list should be mentioned in the cover letter that accompanies your revised manuscript.

In addition to these revisions, you may need to complete some formatting changes, which you will receive in a follow up email. A member of our team will be in touch with a set of requests shortly. If you do not receive a separate email within a few days, please assume that checks have been completed, and no additional changes are required.

We expect to receive your revised manuscript within two weeks.

To submit your revision, please go to https://www.editorialmanager.com/pbiology/ and log in as an Author. Click the link labelled 'Submissions Needing Revision' to find your submission record. Your revised submission must include the following:

- a cover letter that should detail your responses to any editorial requests, if applicable, and whether changes have been made to the reference list

- a Response to Reviewers file that provides a detailed response to the reviewers' comments (if applicable, if not applicable please do not delete your existing 'Response to Reviewers' file.)

- a track-changes file indicating any changes that you have made to the manuscript.

NOTE: If Supporting Information files are included with your article, note that these are not copyedited and will be published as they are submitted. Please ensure that these files are legible and of high quality (at least 300 dpi) in an easily accessible file format. For this reason, please be aware that any references listed in an SI file will not be indexed. For more information, see our Supporting Information guidelines:

https://journals.plos.org/plosbiology/s/supporting-information

*Published Peer Review History*

Please note that you may have the opportunity to make the peer review history publicly available. The record will include editor decision letters (with reviews) and your responses to reviewer comments. If eligible, we will contact you to opt in or out. Please see here for more details:

https://plos.org/published-peer-review-history/

*Press*

Should you, your institution's press office or the journal office choose to press release your paper, please ensure you have opted out of Early Article Posting on the submission form. We ask that you notify us as soon as possible if you or your institution is planning to press release the article.

*Protocols deposition*

To enhance the reproducibility of your results, we recommend that if applicable you deposit your laboratory protocols in protocols.io, where a protocol can be assigned its own identifier (DOI) such that it can be cited independently in the future. Additionally, PLOS ONE offers an option for publishing peer-reviewed Lab Protocol articles, which describe protocols hosted on protocols.io. Read more information on sharing protocols at https://plos.org/protocols?utm_medium=editorial-email&utm_source=authorletters&utm_campaign=protocols

Please do not hesitate to contact me should you have any questions.

Sincerely,

Taylor

Taylor Hart, PhD,

Associate Editor

thart@plos.org

PLOS Biology

Reviewer remarks:

Reviewer #1: I thank the authors for their careful and thorough revision of the manuscript. They have addressed all of my previous comments satisfactorily. I believe the revised manuscript is significantly improved and is suitable for publication.

Reviewer #2: The authors propose that exposure of Drosophila larvae to bacterial uracil activates Duox-dependent oxidative signaling in the gut, which is then detected by a TrpA1-B⁺/Gr66a⁺ neuron in the larval terminal organ, ultimately modifying the adult gustatory response to bacterial peptidoglycan. This is an interesting concept because it links an early microbial experience to long-lasting changes in adult sensory behavior.

Overall, the revision has substantially improved the manuscript. The authors have addressed many of the previous concerns by (1) expanding the figure legends and defining ATB in the main protocol; (2) adding calcium-imaging data from adult ppk23⁺/Gr66a⁻ neurons following larval uracil exposure; (3) testing higher concentrations and repeated exposure to H₂O₂, as well as quinine exposure; (4) adding ROS/H₂O₂ measurements together with a conditioned-medium experiment; (5) incorporating previous literature regarding TrpA1 expression in bitter-sensing neurons; and (6) providing an averaged calcium-response time course while testing cytosine and thymine as related pyrimidines.

Overall, I appreciate the authors' efforts, and several aspects of the manuscript are now considerably stronger. Nevertheless, I believe a few minor issues still deserve attention before publication.

1. The revisions to Figure 1 and the definition of ATB have improved clarity. However, the Figure 1 legend has become unnecessarily long and now includes detailed statistical descriptions that would be more appropriate in the Materials and Methods section. In addition, two statements should be corrected. The phrase "each fly represents an independent experiment" is inaccurate because each fly is an independent observation rather than an independent experiment. Likewise, "Fisher exact t-test" is not a valid statistical test and should be corrected to "Fisher's exact test."

2. Although the measured ROS increase was not directly examined in Duox-knockdown larvae and the precise downstream signaling molecule remains unidentified, I do not consider these limitations sufficient to preclude publication. The current data support the involvement of oxidative signaling, but the manuscript should maintain appropriately cautious language and avoid presenting H₂O₂ as the conclusively identified instructive signal or sole mediator of priming. Increased ROS or hydrogen peroxide is not equivalent to identifying the final biochemical signal responsible for the developmental effect.

3. My remaining concern relates to the strength of the mechanistic conclusions. The title states that "Uracil-Driven ROS signals activate larval TrpA1-B neurons," whereas the Discussion also proposes that ROS may instead generate secondary electrophilic metabolites that ultimately activate TrpA1. These are distinct mechanistic possibilities and should not be presented interchangeably.

More importantly, the newly added experiments show that activating terminal organ (TrpA1⁺) neurons during larval stages with either H₂O₂ or quinine failed to induce adult peptidoglycan aversion, despite confirming neuronal activation. This suggests that activation of these neurons alone is insufficient to account for uracil-dependent priming. Accordingly, the manuscript should either directly test neuronal sufficiency using a selective activation strategy (e.g., optogenetic, thermogenetic, or chemogenetic activation) or revise the title, Highlights, and conclusions to state more explicitly that TrpA1-B neuron activity is necessary but has not been shown to be sufficient, while briefly discussing additional uracil-specific signals that may be required.

More broadly, the current data do not demonstrate a direct synaptic connection between the larval TrpA1-B-positive neuron and the adult ppk23-positive neuronal population, nor do they distinguish among alternative mechanisms such as altered developmental connectivity, receptor expression, neuronal excitability, or broader developmental programming. Therefore, describing the findings as a complete circuit-level mechanism appears somewhat stronger than the available evidence supports.

Finally, the adult ppk23 calcium-imaging data should be described as evidence of altered adult neuronal responsiveness following larval experience.

4. There is a typographical error in which TrpA1 is incorrectly written as TprA1.

Reviewer #3 [Simon Sprecher]: The authors have addressed all points raised, I feel it's an important and interesting contribution.

# Author comment: Author response to Decision Letter 2

**DOI:** 10.1371/journal.pbio.3004042.r005

17 Sep 2026

Attachment Submitted filename: RM et al PB R2 Response to reviewers.docx

# Editor report: Decision Letter 2

**DOI:** 10.1371/journal.pbio.3004042.r006

**Authors:** Taylor Hart, PhD

22 Sep 2026

Dear Dr Royet,

Thank you for the submission of your revised Research Article "Uracil-driven ROS signaling and larval TrpA1-B neuron activation are required for priming the adult Drosophila gustatory response to bacteria" for publication in PLOS Biology. On behalf of my colleagues and the Academic Editor, Piali Sengupta, I am pleased to say that we can in principle accept your manuscript for publication, provided you address any remaining formatting and reporting issues. These will be detailed in an email you should receive within 2-3 business days from our colleagues in the journal operations team; no action is required from you until then. Please note that we will not be able to formally accept your manuscript and schedule it for publication until you have completed any requested changes.

Please take a minute to log into Editorial Manager at http://www.editorialmanager.com/pbiology/, click the "Update My Information" link at the top of the page, and update your user information to ensure an efficient production process.

PRESS

We frequently collaborate with press offices. If your institution or institutions have a press office, please notify them about your upcoming paper at this point, to enable them to help maximise its impact. If the press office is planning to promote your findings, we would be grateful if they could coordinate with biologypress@plos.org. If you have previously opted in to the early version process, we ask that you notify us immediately of any press plans so that we may opt out on your behalf.

We also ask that you take this opportunity to read our Embargo Policy regarding the discussion, promotion and media coverage of work that is yet to be published by PLOS. As your manuscript is not yet published, it is bound by the conditions of our Embargo Policy. Please be aware that this policy is in place both to ensure that any press coverage of your article is fully substantiated and to provide a direct link between such coverage and the published work. For full details of our Embargo Policy, please visit http://www.plos.org/about/media-inquiries/embargo-policy/.

Thank you again for choosing PLOS Biology for publication and supporting Open Access publishing. We look forward to publishing your study.

Sincerely,

Taylor

Taylor Hart, PhD,

Associate Editor

PLOS Biology

thart@plos.org
